# Supplementary material for: Sleep homeostasis regulated by 5HT2b receptor in a small subset of neurons in the dorsal fan-shaped body of drosophila
Source: eLife. 2017 Oct 6;6:e26519. doi: 10.7554/eLife.26519 (PMC5648528; doi:10.7554/eLife.26519)
Supplement: Transparent reporting form [file elife-26519-transrepform.pdf]

eLIFE
1st Floor
24 Hills Road
Cambridge CB2 1JP, UK
P 01223 855340
W elifesciences.org
T @elife

eLife's transparent reporting form

We encourage authors to provide detailed information *within their submission* to facilitate the interpretation and replication of experiments. If you have any questions, please contact us: editorial@elifesciences.org.

Sample-size estimation

- You should state whether an appropriate sample size was computed when the study was being designed
- You should state the statistical method of sample size computation and any required assumptions
- If no explicit power analysis was used, you should describe how you decided what sample (replicate) size (number) to use

Please outline where this information can be found within the submission (e.g., page numbers or figure legends), or explain why this information doesn't apply to your submission:

We stated smaple size and statistical method for every behavioral test.
Figure Legend 1,
Figure Legend 2 (for sleep duration and sleep recovery, respectively)
Figure Legend 5 (for sleep duration and sleep recovery, respectively)
Figure Legend 6,
Figure 1-figure supplement 2,
Figure 1-figure supplement 4,
Figure 2-figure supplement 1,
Figure 5-figure supplement 1 (for sleep duration and sleep recovery, respectively)

eLife Sciences Publications, Ltd is a limited liability non-profit non-stock corporation incorporated in the State of Delaware, USA, with company number 5030732, and is registered in the UK with company number FC030576 and branch number BR015634 at the address 1st Floor, 24 Hills Road, Cambridge CB2 1JP | August 2014

Replicates

- You should report how often each experiment was performed
- You should include a definition of biological versus technical replication
- The data obtained should be provided and sufficient information should be provided to indicate the number of independent biological and/or technical replicates
- If you encountered any outliers, you should describe how these were handled
- Criteria for exclusion/inclusion of data should be clearly stated
- High-throughput sequence data should be uploaded before submission, with a private link for reviewers provided (these are available from both GEO and ArrayExpress)

Please outline where this information can be found within the submission (e.g., page numbers or figure legends), or explain why this information doesn't apply to your submission:

We stated how often experiments were performed on Page 18, Sleep Analysis, Materials and Methods.

Statistical reporting

- Statistical analysis methods should be described and justified
- Raw data should be presented in figures whenever informative to do so (typically when N per group is less than 10)
- For each experiment, you should identify the statistical tests used, exact values of N, definitions of center, methods of multiple test correction, and dispersion and precision measures (e.g., mean, median, SD, SEM, confidence intervals; and, for the major substantive results, a measure of effect size (e.g., Pearson's r, Cohen's d)
- Report exact p-values wherever possible alongside the summary statistics and 95% confidence intervals. These should be reported for all key questions and not only when the p-value is less than 0.05.

Please outline where this information can be found within the submission (e.g., page numbers or figure legends), or explain why this information doesn't apply to your submission:

We stated statistical methods, methods of multiple test correction, dispersion and precision measures, p values for every behavioral test.
Figure Legend 1,
Figure Legend 2 (for sleep duration and sleep recovery, respectively)
Figure Legend 5 (for sleep duration and sleep recovery, respectively)
Figure Legend 6 c
Figure 1-figure supplement 2 , Figure 1-figure supplement 4 , Figure 2-figure supplement 1 , Figure 5-figure supplement 1 (for sleep duration and sleep recovery, respectively)

(For large datasets, or papers with a very large number of statistical tests, you may upload a single table file with tests, Ns, etc., with reference to page numbers in the manuscript.)

Additional data files ("source data")

- We encourage you to upload relevant additional data files, such as numerical data that are represented as a graph in a figure, or as a summary table
- Where provided, these should be in the most useful format, and they can be uploaded as "Source data" files linked to a main figure or table
- Include model definition files including the full list of parameters used
- Include code used for data analysis (e.g., R, MatLab)
- Avoid stating that data files are "available upon request"

Please indicate the figures or tables for which source data files have been provided:

The submission included Movies (line 169, 173, 194), but no additional data files.
